# Risk Culture and COVID-19 Protective Behaviors: A Cross-Sectional Survey of Residents in China

**DOI:** 10.3389/fpubh.2021.686705

**Published:** 2021-11-01

**Authors:** Xuejing Bi, Qiao Zhang, Kaisheng Fan, SiYu Tang, HanWen Guan, XueQin Gao, Yu Cui, Yi Ma, QunHong Wu, YanHua Hao, Ning Ning, Chaojie Liu

**Affiliations:** ^1^School of Health Management, Harbin Medical University, Harbin, China; ^2^School of Psychology & Public Health, La Trobe University, Melbourne, VIC, Australia

**Keywords:** risk culture, COVID-19, protective behaviors, risk perception, knowledge, trust

## Abstract

The COVID-19 outbreak caused by the Severe Acute Respiratory Syndrome CoronaVirus type 2 (SARS-CoV-2) has spread across the world. However, our understanding of the public responses, in particular in adopting protective behaviors, has been limited. The current study aimed to determine the level of protective behaviors adopted by the residents in China and its association with their cultural attributes. A national cross-sectional online survey was conducted in mainland China from 4^th^ to 13^th^ August 2020. Protective behaviors were assessed as a summed score (ranging from 0 to 40) measured by ten items. The self-report tendency of study participants toward the four cultural attributes (individualism, egalitarianism, fatalism, hierarchy) was rated on a seven-point Likert scale. A total of 17651 respondents returned a valid questionnaire, representing 47.9% of those who accessed the online survey. Most (89.8%) respondents aged between 18 and 45 years in the age range of and 47.7% were male. High levels of protective behaviors (34.04 ± 5.78) were reported. The respondents had high scores in the cultural attributes of hierarchy (Median = 5) and egalitarianism (Median = 5), compared with low scores in individualism (Median = 1) and fatalism (Median = 1). High levels of protective behaviors were associated a higher tendency toward egalitarianism (AOR = 2.90, 95% CI 2.67–3.15) and hierarchy (AOR = 1.66, 95% CI 1.53–1.81) and a low tendency toward fatalism (AOR = 1.79, 95% CI 1.63–1.97) and individualism (AOR = 2.62, 95% CI 2.41–2.85). The cultural attributes explained 17.3% of the variations in the protective behavioral scores. In conclusion, the adoption of protective behaviors is associated a risk culture characterized by high levels of hierarchy and egalitarianism and low levels of individualism and fatalism. Government actions and communication strategies need to adapt to the cultural characteristics of their target audience.

## Introduction

In December 2019, SARS-CoV-2, a new coronavirus strain, was reported to infect human beings, resulting in severe respiratory illness COVID-19. Compared with MERS and SARS, COVID-19 has spread more rapidly (1). The World Health Organization (WHO) declared the COVID-19 outbreak as a public health emergency of international concern (1, 2). To date, the global outcome has amounted to over 109 million confirmed cases and more than 2.4 million deaths (https://covid19.who.int/table). The most critical transmission route of SARS-CoV-2 is human-to-human via respiratory droplets and direct contacts (3). Although vaccines have been developed, the global pandemic is far from over (4). Non-pharmacological interventions, including protective behaviors such as hand hygiene, social distancing, mask-wearing, movement restriction, and public compliance with testing, contact tracing, and quarantine requests remain to be critical in the battle against COVID-19 (5–7).

Despite strong advocacy from the WHO, the public endorsement of the protective behaviors vary considerably across regions and countries (8–12). Empirical evidence shows that the public endorsement, or otherwise, of the protective behaviors can be shaped by many factors such as the socio-demographic characteristics of people and their access to knowledge and information, risk perceptions, and emotions (11, 13, 14). Differences in the public protective behaviors may be better described under specific cultural contexts (2, 15, 16). The concept of culture delineates a group of people's consciousness and the modalities of their actual behaviors (7, 12, 17, 18). However, our understanding of the cultural impact on the public responses to COVID-19 has been limited.

**Figure 1:**
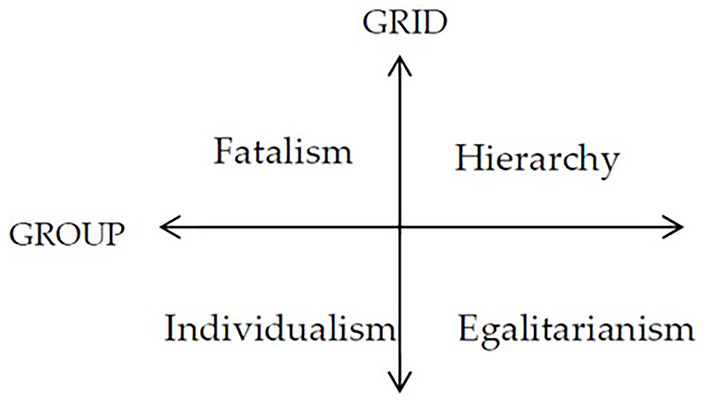
The group-Grid framework.

Culture is one of the most widely used terms in social science despite a lack of consensus on its measurements (19). The cultural theory holds that culture is reflected by how people think and behave (20–23). Douglas used a “Grid-Group” framework to describe individual tendency toward various cultural attributes. Dake further revised this framework and developed a Cultural Biases Questionnaire (24). The questionnaire contains four quadrants divided by a group dimension and a grid dimension. The group dimension refers to the extent that a group binds a person. A high sense of belonging to a group (“us” vs. “them”) entails collectivism and encourages cooperation. The grid dimension refers to the extent to which relations are prescribed. A higher grid indicates higher acceptance of prescribed behaviors (21, 25–27). Four quadrants of cultural attributes arise from the two dimensions: individualism (low sense of group belonging and low acceptance of prescribed behaviors); fatalism (low sense of group belonging and high acceptance of prescribed behaviors); hierarchy (high sense of group belonging and high acceptance of prescribed behaviors); egalitarianism (high sense of group belonging and low acceptance of prescribed behaviors) (28). The Dake's questionnaire provided an ideal framework for the purpose of our study (Figure 1). Empirical evidence shows that public behaviors are often constrained by these cultural attributes (21). We hypothesized that the four quadrants of cultural attributes were associated with the behavioral choice of the public in response to the outbreak of COVID-19.

There has been a consensus that individualism has a detrimental effect on collective actions due to its self-directed interest (29). Those with a fatalism view does do not believe that they have any control over their destiny, which can disempower them from making a contribution to social goods (21, 30). By contrast, those who adhere to egalitarian values believe that everyone in their society is equal (21, 23). They tend to pursue the common interest of their groups (31), which may translate into a high level of compliance and adoption of COVID-19 protective behaviors. Similarly, a hierarchical culture encourages everyone to follow instructions to safeguard their status and interests (32). The individuals following a hierarchical culture tend to trust experts and authorities (21). By contrast, those who prefer an individualistic culture are more concerned about individual freedom. They tend to have high tolerance to public risks (21, 32).

The national culture of China is often described as one with a hierarchical structure and low levels of individualism (32, 33). Commentaries often link the successful containment of the outbreak of COVID-19 in China with its strong governmental power resulting from the centralized and one-party system (34, 35). However, limited attention has been paid to how the public responded. Our study at the very early stage of the COVID-19 outbreak in China revealed a high percentage (71%) of embracement of the protective behaviors prescribed by the authorities (34). It is important to note that unlike in many other countries, the Chinese government used very little, if any, the financial incentives (and penalty) to enforce the restrictions (28). Instead, local community organizations, neighborhoods and employers were mobilized to mount public pressures. This approach aligns well with the collectivism cultural attributes (28), but forms a sharp contrast with the strategies adopted in the western countries where individual freedom is highly prioritized. In those countries, police are usually supposed to enforce the public orders and a fine is often imposed to deter individuals from violating the public orders. It is reasonable to assume that the collectivism cultural attributes may present an opportunity for an alternative approach to the public compliance with the restrictive measures in the absence of strong policing and penalty measures (21).

The objectives of this study included two folds. First, we investigated the level of public endorsement of the self-report protective behaviors seven months after the initial outbreak of COVID-19 in comparison with the findings of our previous study at the early stage of the outbreak. Second, we tested the hypotheses of the associations between cultural attributes and protective behaviors. Although the Group-Grid cultural framework has been widely applied in many areas of studies (e.g., human behaviors on environmental concerns, public goods, and politics) (12, 20), its use in the context of the global Covid-19 pandemic has been limited (36). The study addressed the gap in the literature.

## Methods

An online cross-sectional survey was conducted in China. Ethics approval for the study protocol was obtained from the Ethics Committee of Harbin Medical University (IRB number HMUIRB202000004). Implied informed consent was sought from all participants before the start of the survey.

### Study Participants and Data Collection

Data were collected online from 4^th^ to 13^th^ August 2020 via Wenjuanxing, a widely accepted online questionnaire survey platform in China. Those who were older than 18 years were eligible to participate in this survey. Potential participants were invited to read and agree with the informed consent statements before proceeding to the survey. Each IP address was allowed to submit one questionnaire only. Participation in the survey was anonymous. Respondents could withdraw at any time before submitting the questionnaire, but not afterward due to the anonymous nature of the survey.

Local community health services across the 31 provinces in mainland China were asked to help disseminate the survey to their local residents through a weblink or a QR code. Those who participated in the survey were also encouraged to circulate the survey invitation in their WeChat social media groups. In total, the online survey platform recorded 36,862 responses. Our pilot test indicated that the survey would take at least 10 min to complete. Therefore, the responses (*n* = 17,623) submitted within 8 min were excluded. We also conducted a logic check using the questions with inherent logic connections. For example, a respondent who often “communicated with family and friends about the epidemic, both online and offline” is unlikely to rarely “communicate with family and friends, both online and offline, during the epidemic?” The logic audit identified 1588 returned questionnaires containing logic errors. This resulted in a final sample of 17,651 (47.9% of returned questionnaires) for data analyses.

### Measures

#### Outcome Variable

Protective behaviors were the primary interest of this study. Respondents were asked to report their compliance with ten behavioral items (e.g., hand hygiene, social distancing, face mask, etc.) prescribed by the Centers for Disease Prevention and Control over the past one month on a five-point Likert scale ranging from 0 “never” to 4 “always”. These items are commonly adopted protective behaviors during a pandemic according to the review conducted by Bish and Michie (6). The ten behavioral items were identified in line with the governmental guidelines in China and Bish's study A summed score was calculated (ranging from 0 to 40), with a higher score indicating a higher level of self-report protective behaviors.

#### Exposure Variable

Cultural attributes served as the exposure variable tested in this study. Each of the quadrants (individualism, fatalism, hierarchy, egalitarianism) of cultural attributes was were assessed against the following three questions: “What motivated you to take protective actions”; “What are the main reasons for the COVID-19 outbreak”; and “How did you feel toward COVID-19” (Supplementary Table 1) (24). These questions were developed based on the existing literature (19, 21, 29, 37) and were adapted to the COVID-19 context. One answer to each question corresponding to each cultural quadrant was assigned, considering both the value of the cultural worldview (32) and the country context (19). Respondents were asked to rate their agreement with each assigned answer concerning the three questions. A summed score for each of the cultural quadrants was calculated, with a higher score indicating a higher tendency toward the respective cultural attribute.

#### Control Variables

Many factors can influence human behaviors. This study chose the rational choice model (RCM) and the knowledge, attitude, practice (KAP) model to guide the selection of independent variables because they are highly relevant to the explanation of individual behaviors that may have a significant impact on the public (10, 11, 38, 39). Under the context of the outbreak of COVID-19, individuals need to make a quick behavioral choice under tremendous public pressures in a collectivist culture. The RCM is aligned with the circumstance very well as it adopts the concepts of rational actors, self-interest, and the invisible hand (40, 41). Meanwhile, there is strong empirical evidence to support the KAP framework. For example, misinformation and disinformation have attracted increasing concerns in the international community on their role in misguiding people's behaviors in response to the outbreak of COVID-19 (34). Social marketing and health education campaigns have been focused on improving knowledge and attitudes of the public. Furthermore, there has been increasing recognition of social determinants of health behaviors (40, 41). The control variables measured in this study included:

#### Socio-Demographic Characteristics

Data in relation to age (<30, 30–39, ≥40 year), gender (male vs. female), marital status (married vs. others), religion (yes vs. no), educational attainment (with vs. without tertiary qualification), and residency (rural vs. urban) were captured in the survey. Human behaviors may vary by these characteristics (34).

#### Knowledge

Knowledge is commonly considered as a prerequisite condition for enabling the public to take action (11). The knowledge test embedded in this study derived from the list of knowledge sets promoted by the National Health Commission (http://www.nhc.gov.cn/) and the Chinese Centers for Disease Control and Prevention (http://www.chinacdc.cn/jkzt/crb/zl/szkb_11803/) in line with the WHO guidelines (34). It covered the nature of COVID-19, its transmission routes, sanitation measures, and preventive strategies (34). A score of 1 was assigned to a correct answer, 0 otherwise. This resulted in a summed score ranging from 2 to 21 for each respondent. High levels of knowledge were assumed for those who achieved a score above the mean value.

#### Trust

Trust plays a critical role in the public acceptance of information and advice from the government (42). In this study, respondents were asked to rate their trust with the sources of information coming from the international agencies (WHO and the United Nations), the Chinese government, and the Chinese scientists (for example, Dr. Nanshan Zhong), respectively, on a five-point Likert scale ranging from 0 “never” to 4 “always”. A summed score was calculated, which ranged from 0 to 12, with a higher score indicating a higher level of trust. Those with a summed score above 9 were deemed with high trust in others.

#### Risk Perception

Risk perception affects behaviors through direct or indirect avenues (10, 18, 34, 43). The risk perception scale developed by the research team in 2018 was used in this study (34). The instrument demonstrated good reliability (Cronbach's α = 0.824) and construct validity in CFA (GFI = 0.982, AGFI = 0.961, IFI = 0.972, RMSEA = 0.062). It measures three components of risk perception: susceptibility (3 items), severity (3 items), and controllability (3 items). Respondents were asked to rate their perceptions on a six-point Liker scale, ranging from 0 “strongly disagree” to 5 “strongly agree”. A summed score was calculated for each component (ranging from 0 to 15), with a score above 9 indicating a high level of risk perception.

### Statistical Analysis

The socio-demographic characteristics of respondents were described through frequency distributions for categorical and ordinal data, mean values and standard deviations (SDs) for continuous data with a normal distribution, and medians and interquartile ranges (IQRs) for continuous data with a non-normal distribution.

The protective behavioral scores were severely positively biased. Therefore, they were transformed into two categories using the mean value as a cutoff point: high (>34.04) vs. low (≤34.04). We used Chi-square to test the statistical differences of protective behaviors in the respondents with different characteristics.

Responses to the cultural quadrants were also extremely biased and therefore collapsed into a smaller number of categories (Supplementary Table 2) for the purpose of statistical modeling. Multivariate logistic regression models were established to determine the associations between cultural attributes and self-report protective behaviors after adjustment for variations in the control variables. The regression model inclusive of the cultural attributes was compared with that exclusive of the cultural attributes. The difference in the R^2^ of the two models (ΔR^2^) indicates the percentage contribution of the cultural attributes in explaining the variations of protective behaviors. To test the robustness of the logistic regression models, we also performed linear regression analyses were established with the protective behavior scores being treated as a continuous variable. The results are consistent with those of the logistic regression models (Supplementary Table 3).

All data analyses were performed using the SPSS statistic software version 23.0 (IBM). A two-sided *p*-value < 0.05 was considered statistically significant.

## Results

### Characteristics of Respondents

The respondents had a mean age of 30.55 (SD = 9.8) years: about 90% were younger than 45. Slightly less than half of the respondents were men (47.7%) and resided in rural areas (40.8%). The majority (68.4%) of respondents obtained a tertiary qualification. Less than a quarter (24.0%) reported a religious belief (Table 1).

**Table 1:** Characteristics of study participants (*n* = 17,651).

**Characteristics**		**Sample size**	**High protective behaviors (>34.04)**	*****χ^2^*****	***P***
		***n* (%)**	***n* (%)**		
Gender	Female	9,227 (52.3)	5,342 (57.9)	68.07	<0.001
	Male	8,424 (47.7)	4,356 (51.7)		
Residence	Rural	7,195 (40.8)	3,735 (51.9)	45.11	<0.001
	Urban	10,456 (59.2)	5,963 (57.0)		
Marital status	Married	7,837 (44.4)	3,883 (49.5)	165.79	<0.001
	Others	9,814 (55.6)	5,815 (59.3)		
Age (years)	<30	9,110 (51.6)	4,659 (51.1)	111.91	<0.001
	30–39	5,573 (31.6)	3,319 (59.6)		
	≥40	2,968 (16.8)	1,720 (58.0)		
Tertiary Education	No	5,583 (31.6)	2,993 (53.6)	5.87	0.015
	Yes	12,068 (68.4)	6,705 (55.6)		
Religion	No	13,418 (76.0)	7,389 (55.1)	0.35	0.553
	Yes	4,233 (24.0)	2,309 (54.5)		
Knowledge score	<17.7 (low)	6,145 (34.8)	2,890 (47.0)	238.44	<0.001
	≥17.7 (high)	11,506 (65.2)	6,808 (59.2)		
Trust score	<9 (low)	746 (4.2)	155 (20.8)	367.28	<0.001
	≥9 (high)	16,905 (95.8)	9,543 (56.5)		
Perceived severity	<9 (low)	8,286 (46.9)	4,444 (53.6)	10.83	0.001
	≥9 (high)	9,365 (53.1)	5,254 (56.1)		
Perceived susceptibility	<9 (low)	15,771 (89.3)	8,709 (55.2)	4.64	0.031
	≥9 (high)	1,880 (10.7)	989 (52.6)		
Perceived controllability	<9 (low)	9,341 (52.9)	5,035 (53.9)	8.68	0.003
	≥9 (high)	8,310 (47.1)	4,663 (56.1)		
Egalitarianism	0,1,2,3,4 (L)	7,998 (45.3)	3,360 (42.0)	1,416.29	<0.001
	5 (M)	4,164 (23.6)	2,233 (53.6)		
	6,7 (H)	5,489 (31.1)	4,105 (74.8)		
Hierarchy	0,1,2,3,4 (L)	6,316 (35.8)	2,670 (42.3)	1,058.63	<0.001
	5 (M)	4,846 (27.5)	2,467 (50.9)		
	6,7 (H)	6,489 (36.8)	4,561 (70.3)		
Individualism	0 (L)	4,836 (27.4)	3,664 (75.8)	1,467.72	<0.001
	1,2 (M)	3,767 (21.3)	2,219 (58.9)		
	3,4,5,6,7 (H)	9,048 (51.3)	3,815 (42.2)		
Fatalism	0 (L)	4,670 (26.5)	3,270 (70.0)	584.76	<0.001
	1,2 (M)	8,389 (47.5)	4,188 (49.9)		
	3,4,5,6,7 (H)	4,592 (26.0)	2,240 (48.8)		

The respondents displayed a high level of knowledge about COVID-19, with a mean score of 17.86 (SD = 2.99). About 65.2% obtained a score above the mean value. The vast majority (95.8%) were deemed to have high (≥9) trust in others. Around half of respondents perceived high risk in severity (53.1%) and controllability (47.1%) of COVID-19; whereas, only 10.7% perceived high risk of susceptibility (Table 1).

### Cultural Attributes

**Figure 2:**
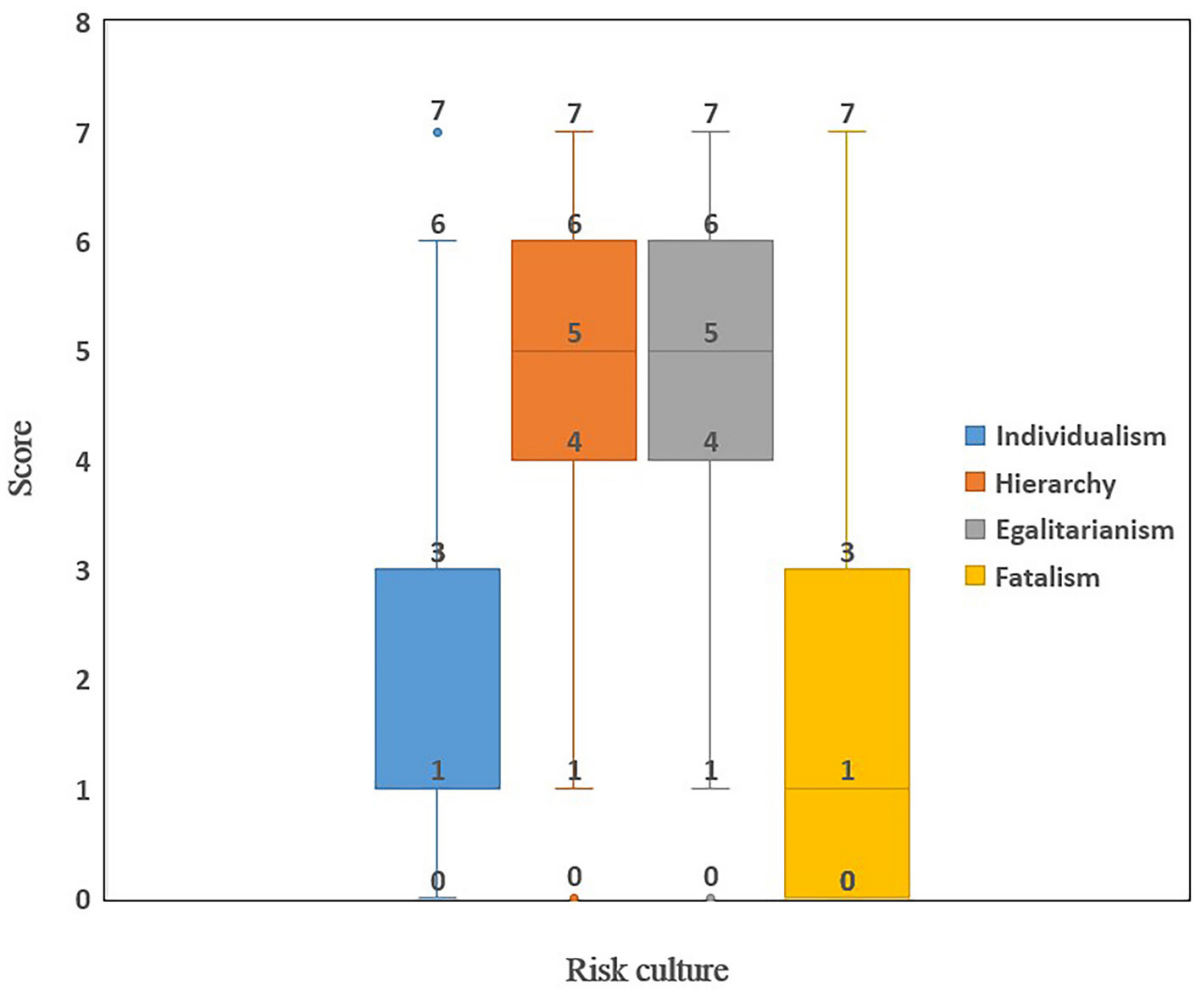
Status quo of culture type.

High scores in egalitarianism (Median = 5) and hierarchy (Median = 5) were found in the respondents, compared with low scores in individualism (Median = 1) and fatalism (Median = 1). More than 80% of respondents reported a score above 4 for hierarchy (87.1%) and egalitarianism (80.5%). By contrast, less than 20% of respondents reported a score above 4 for individualism (17.9%) and fatalism (12.3%) (Figure 2).

### Protective Behaviors

The respondents had a mean behavioral score of 34.04 (SD = 5.78): 54.9% were deemed as having a high level (>34.04) of protective behaviors. The vast majority reported at least some compliance (≥3) with the prescribed protective behaviors: more than 90% followed official advice (93.8%), kept social distance (93.4%), and maintained good ventilations (94.4%). The least compliant tasks were crowd avoidance (77.1%) and staying at home (76.1%) (Table 2).

**Table 2:** Protective behaviors endorsed by respondents.

**Behavior**	**Mean**	**Standard deviation**	**Min**	**Max**	**% (≥3)**
Follow official advice	3.65	0.69	0.0	4.0	93.8
Keep social distance	3.58	0.70	0.0	4.0	93.4
Avoid sharing food utensils	3.45	0.93	0.0	4.0	87.3
Avoid public transport	3.29	0.95	0.0	4.0	81.3
Maintain good ventilation inside of buildings	3.64	0.67	0.0	4.0	94.4
Practice hand hygiene	3.33	0.92	0.0	4.0	82.4
Maintain good nutrition and physical activities	3.37	0.79	0.0	4.0	87.0
Wear face mask	3.40	0.85	0.0	4.0	86.1
Avoid crowd	3.19	0.97	0.0	4.0	77.1
Stay at home	3.13	1.00	0.0	4.0	76.1
Total score	34.04	5.78	0.0	40.0	81.0(≥30)

### Factors Associated With Protective Behaviors

Female respondents were more likely to adopt protective behaviors. Those who were married, obtained a tertiary qualification, and resided in rural areas reported higher levels of protective behaviors (*p* <0.001). Better knowledge, higher trust, and higher risk perceptions were associated with higher levels of protective behaviors (*p* < 0.01) (Table 1).

The four quadrants of cultural attributes were associated with protective behaviors after adjustment for variations in the control variables. The hypotheses were supported: egalitarianism (AOR = 2.90, 95% CI 2.67–3.15) and hierarchy (AOR = 1.66, 95% CI 1.53–1.81) were positively associated with protective behaviors; whereas, fatalism (AOR = 1.79, 95% CI 1.63–1.97) and individualism (AOR = 2.62, 95% CI 2.41–2.85), were negatively associated with protective behaviors. The inclusion of the cultural attributes increased the R^2^ of the regression models significantly. The cultural attributes explained 17.3% of the variations of the protective behaviors (Table 3).

**Table 3:** Predictors of protective behaviors–results from logistic regression models.

**Predictor**		**Reference**			**Model one**		**Model two**	
			***Unadjusted***		***Adjusted***		***Adjusted***	
			**OR (95% CI)**	***P***	**OR (95% CI)**	***P***	**OR (95% CI)**	***P***
**Cultural attributes**								
Individualism	M	H	4.29 (3.97 to 4.64)	<0.001	_	_	2.62 (2.41 to 2.85)	<0.001
	L		1.97 (1.82 to 2.12)	<0.001	_	_	1.55 (1.43 to 1.68)	<0.001
Egalitarian	M	L	1.60 (1.48 to 1.72)	<0.001	_	_	1.37 (1.26 to 1.48)	<0.001
	H		4.09(3.80 to 4.42)	<0.001	_	_	2.90 (2.67 to 3.15)	<0.001
Hierarchy	M	L	1.42 (1.31 to 1.53)	<0.001	_	_	1.11 (1.02 to 1.21)	0.013
	H		3.23 (3.00 to 3.48)	<0.001	_	_	1.66 (1.53 to 1.81)	<0.001
Fatalism	M	H	2.45 (2.25 to 2.67)	<0.001	_	_	1.79 (1.63 to 1.97)	<0.001
	L		1.05 (0.97 to 1.13)	0.213	_	_	1.05 (0.97 to 1.14)	0.268
**Control variables**								
Gender	Female	Male	1.28 (1.21 to 1.36)	<0.001	1.24 (1.16 to 1.315)	<0.001	1.34 (1.25 to 1.43)	<0.001
Residency	Urban	Rural	1.23 (1.16 to 1.31)	<0.001	1.13 (1.06 to 1.20)	<0.001	1.13 (1.06 to 1.21)	<0.001
Marital status	Married	Others	1.48 (1.40 to 1.57)	<0.001	1.34 (1.26 to 1.43)	<0.001	1.23 (1.15 to 1.32)	<0.001
Age (years)	30-39	≥40	1.41 (1.32 to 1.51)	<0.001	_	_		_
	<30		1.32 (1.21 to 1.43)	<0.001	_	_		_
Tertiary education	Yes	No	1.08 (1.02 to 1.15)	0.015	_	_		_
Perceived severity	High	Low	1.11 (1.04 to 1.17)	0.001	1.19 (1.11 to 1.27)	<0.001	1.08 (1.00 to 1.16)	0.030
Perceived susceptible	Low	High	1.11 (1.01 to 1.22)	0.031	1.12 (1.01 to 1.24)	0.027	1.04 (0.93 to 1.16)	0.550
Perceived controllability	High	Low	1.09 (1.03 to 1.16)	0.003	1.13 (1.06 to 1.21)	<0.001	1.17 (1.09 to 1.26)	<0.001
Knowledge	≥17.7 (High)	<17.7 (Low)	1.63 (1.53 to 1.74)	<0.001	1.45 (1.36 to 1.55)	<0.001	1.29 (1.20 to 1.38)	<0.001
Trust	≥9 (High)	<9 (Low)	4.94 (4.13 to 5.91)	<0.001	4.48 (3.73 to 5.37)	<0.001	2.59 (2.14 to 3.15)	<0.001
*R^2^*(%)					5.7		23.0	
Δ*R^2^*(%)							17.3	

## Discussion

Overall, a high level of protective behaviors was reported in this study as indicated by the high compliance of respondents with official advice (93.8%), ventilation (94.4%), and social distancing (93.4%). The least compliant tasks in ration to crowd avoidance and staying at home were also received over 76% compliance. These results are consistent with the findings of other studies, such as the medical students in Iran (10, 11, 34, 44). Compared with the results of our study at the early stage of the COVID-19 outbreak, there was a clear tendency of increased social gathering and use of public transport, possibly due to the relaxation of restrictive measures (6, 34).

This study confirmed that cultural attributes are significant predictors of protective behaviors. We found that the cultural attributes could explain 17.3% of the variations of the protective behaviors. The cultural attributes of the study participants were characterized by a high level of egalitarianism and hierarchy and a low level of individualism and fatalism. All of the cultural attributes were significantly associated with self-report protective behaviors, with AORs ranging from 1.05 to 2.90. The results are similar to those of Zeng's study, in which cultural attributes were found to be associated with pro-environmental behaviors (21, 28). Previous studies suggest that culture functions as an orienting mechanism, which may help people to navigate through the world full of uncertainties and risks (38). The culture theory proposes that risks are “socially selected and at least in part socially constructed” (19). Cultural contexts can constrain the development of the core values and behavioral preferences of individuals, leading to a conscious or unconscious bias toward risks and risk behaviors (19, 45). In a hierarchical society, people are willing to follow the rules and procedures of authorities, which are usually guided by the egalitarian principles (protecting the vulnerable) (19). Egalitarianism and hierarchies foster a high level of collective thinking (19, 21). By contrast, an individualist culture embraces acts on of self-interest, although it can be context-dependent (32). A fatalistic approach usually involves specific coping strategies that avoid confrontations with risks (8).

Consistent with previous studies, protective behaviors were also found to be associated with individual characteristics of the study participants (46). Women, urban dwellers, and married couples are more likely to embrace prescribed behaviors than others. Some researchers argued that this is perhaps a reflection of felt vulnerability and a sense of responsibility (6, 21, 34). No doubt Clearly, protective behaviors can also be shaped by knowledge and perceptions of risks, which are usually the primary focus of educational campaigns (10, 11, 34, 43). However, it is important to note that these variables all had a small adjusted odds ratio (AOR <2) and collectively explained a very small percentage of the variations in protective behaviors according to our modeling, far less than that explained by the cultural attributes.

Trust plays a critical role in risk communication and educational campaigns in response to public health emergencies (6, 23, 32). Indeed, trust was proved to be a significant predictor of protective behaviors (9). However, the AOR of trust declined from 4.94 to 2.59 after the cultural attributes were introduced into the regression models. Trust affects the credibility of messages conveyed by the messengers (6). High levels of trust are often embedded in the culture characterized by egalitarianism and hierarchy (19, 21), which can facilitate public participation and joint efforts in public health emergency responses (47).

A better understanding of how individual behaviors are rooted in one's cultural experiences can help with the better design of governmental and professional interventions (12). Different communication and education strategies should apply to the people with various cultural attributes (2). Policies that are aligned well with local cultural values are much easy to be understood and accepted easier for people to understand and accept (38). The COVID-19 pandemic has highlighted the great importance of public participation. A centralized and authoritarian approach appears to work well in the cultural context of egalitarianism and hierarchy. Meanwhile, individualism and fatalism have been proved to be detrimental to public responses to the pandemic. The experiences of some countries have demonstrated the lack of effectiveness of voluntary measures under such cultural contexts (45). Clearly, there is a need to re-examine the role and functions of the government (48). Nevertheless, the principles of effective communication strategies remain unchanged (34), which require openness and honesty. Effective communication can help build public trust and confidence in the authorities (6, 49).

### Strength and Limitation

To our knowledge, this is the first study of its kind under the context of COVID-19 (2, 10, 11, 39, 50). The sample size of this study is large, with participants coming from nationwide in mainland China. However, the study also has several limitations. First, the survey was conducted online, and the sample was biased toward the young and those with higher educational qualifications. Second, the study adopted a cross-sectional design. No causal relationships should be assumed. Third, the study used attitudinal questions to measure cultural attributes, and is subject to the common problems of subjective measurements. Finally, the nature of the study design prevented us from exploring the dynamics of interaction between the government and the public. Further studies with a transcultural comparison focus are warranted.

### Implications and Contribution

Public mobilization and participation are essential in the battle against COVID-19. The effectiveness, or otherwise, of governmental interventions can be determined by how the public respond to the interventions. This study proved that the cultural attributes are associated with self-report protective behaviors. The results have significant implications for on the development of public health emergency strategies. The potential detrimental effects associated with individualism and fatalism need to be managed appropriately.

## Data Availability Statement

The raw data supporting the conclusions of this article will be made available by the authors, without undue reservation.

## Ethics Statement

The studies involving human participants were reviewed and approved by the Ethics Committee of Harbin Medical University. IRB code is HMUIRB20200004. The patients/participants provided their written informed consent to participate in this study.

## Author Contributions

YH and NN took overall responsibility for the study design, coordination of the survey, development of the analysis framework, and writing. XB, QZ, and KF participated in the research design, conducted the survey and data analyses, and drafted the manuscript. ST, HG, and XG participated in the design of the research and revision of the manuscript. YC and YM participated in the literature review and data collection. QW and CL supervised the data analyses, interpreted the results, and revised the manuscript. All authors have read and approved the final manuscript.

## Funding

This study was funded by National Natural Science Foundation of China (NSFC) (71673072 and 72042001). The funding body did not influence study design, data collection, data analysis, data interpretation, or writing the manuscript.

## Conflict of Interest

The authors declare that the research was conducted in the absence of any commercial or financial relationships that could be construed as a potential conflict of interest.

## Publisher's Note

All claims expressed in this article are solely those of the authors and do not necessarily represent those of their affiliated organizations, or those of the publisher, the editors and the reviewers. Any product that may be evaluated in this article, or claim that may be made by its manufacturer, is not guaranteed or endorsed by the publisher.
