# Supplementary material for: Risk Culture and COVID-19 Protective Behaviors: A Cross-Sectional Survey of Residents in China
Source: Front Public Health. 2021 Nov 1;9:686705. doi: 10.3389/fpubh.2021.686705 (PMC8592257; doi:10.3389/fpubh.2021.686705)
Supplement: Supplementary file 1 [file Table_1.DOCX]

**eTable 1 Cultural attributes measured in the Group-Grid framework**

| Question | Cultural Quadrant | Attributes | Scoring |
| --- | --- | --- | --- |
| What motivated you to take protective actions | Hierarchy | Laws and rules | 0=Not at all  1=A little  2=Generally  3=A great deal |
|  | Egalitarianism | Everyone around me |  |
|  | Individualism | No cost on my freedom or financial rights |  |
|  | Fatalism | Irrelevant and not motivated |  |
| What are the main reasons for the COVID-19 outbreak | Hierarchy | Ineffective governmental interventions | 0=No  1=Yes |
|  | Egalitarianism | Public complacence |  |
|  | Individualism | Inadequate personal protections |  |
|  | Fatalism | Misfortune and bad luck |  |
| How did you feel towards COVID-19 | Hierarchy | Not much need for governmental interventions (reversed item) | 0=Disagree  1=Generally disagree  2=Generally agree  3=Agreement |
|  | Individualism | Individuals should be subordinated to collective interests (reversed item) |  |
|  | Egalitarianism | Everyone needs to be protected |  |
|  | Fatalism | Plague is God's arrangement, a kind of destiny |  |

**eTable 2 Coding of variables**

| Variables | Value assignments | Mean/IQRs | Std | Min | Max |
| --- | --- | --- | --- | --- | --- |
| Protective Behaviors | Low ≤34.04 =0  High >34.04 =1 | 34.04 | 5.78 | 0 | 40 |
| Culture | Hierarchy  0,1,2,3,4=1;5=2;6,7=3 | IQR(5,6-4) | - | 0 | 7 |
|  | Egalitarian  0,1,2,3,4=1;5=2;6,7=3 | IQR(5,6-4) | - | 0 | 7 |
|  | Individualism  0= 1; 1,2= 2 ; 3,4,5,6,7= 3 | IQR(1,3-1) | - | 0 | 7 |
|  | Fatalism  0= 1 ; 1,2= 2 ; 3,4,5,6,7= 3 | IQR(1,3-0) | - | 0 | 7 |
| Gender | Male=0，Female=1 | - | - | 0 | 1 |
| Marry | Others=0，Married=1 | - | - | 0 | 1 |
| Aged | Low <30=1 Middle 30-40=2 High≥40=3 | 30.55 | 9.80 | 18 | 99 |
| Education | Bellow bachelor=0  Bachelor & above=1 | - | - | 0 | 1 |
| Residence | Rural =0，urban =1 | - | - | 0 | 1 |
| Religion | NA=0，Yes=1 | - | - | 0 | 1 |
| Knowledge | Low <‾X =0 High≥‾X =1 | 17.86 | 2.99 | 3 | 21 |
| Trust | Low ≤ 9 =0 High＞9 =1 | 13.31 | 1.79 | 3 | 15 |
| Risk  perception | Perceived severity  Low <9 = 0 High≥ 9 = 1 | 8.49 | 3.93 | 0 | 15 |
|  | Perceived controllability  Low <9 = 0 High≥ 9 = 1 | 8.61 | 3.58 | 0 | 15 |
|  | Perceived susceptibility  Low <9 = 0 High ≥ 9 = 1 | 3.75 | 3.50 | 0 | 15 |

**eTable 3: Predictors of protective behaviors – results of linear regression models**

| **Predictor** | **Model One** | |  |  | **Model Two** | |  |
| --- | --- | --- | --- | --- | --- | --- | --- |
|  | Beta | T | p |  | Beta | T | p |
| **Cultural attributes** |  |  |  |  |  |  |  |
| Individualism | - | - |  |  | -0.19 | -25.21 | <.001 |
| Egalitarian | - | - |  |  | 0.19 | 25.70 | <.001 |
| Hierarchy | - | - |  |  | 0.10 | 12.57 | <.001 |
| Fatalism | - | - |  |  | -0.09 | -11.72 | <.001 |
| **Control variables** | - | - |  |  |  |  | <.001 |
| Gender | -0.05 | -6.88 | <.001 |  | -0.06 | -8.58 | <.001 |
| Residency | 0.03 | 3.86 | <.001 |  | 0.03 | 3.77 | <.001 |
| Marital status | 0.07 | 7.36 | <.001 |  | 0.05 | 6.00 | <.001 |
| Age | -0.00 | -.43 | .667 |  | -0.02 | -2.24 | .025 |
| Education | -0.01 | -1.13 | .260 |  | -0.02 | -2.19 | .029 |
| Severity | 0.04 | 4.93 | <.001 |  | 0.01 | 1.68 | .093 |
| Susceptible | -0.02 | -2.20 | .028 |  | 0.00 | 0.16 | .874 |
| Controllable | 0.03 | 3.72 | <.001 |  | 0.03 | 4.44 | <.001 |
| Knowledge | 0.09 | 11.38 | <.001 |  | 0.05 | 7.03 | <.001 |
| Trust | 0.13 | 17.72 | <.001 |  | 0.07 | 9.57 | <.001 |
| F | 77.82 | |  |  | 256.02 | |  |
| R^2^ (%) | 4.2 | |  |  | 16.8 | |  |
